# Cellular senescence and wound healing in aged and diabetic skin

**DOI:** 10.3389/fphys.2024.1344116

**Published:** 2024-02-19

**Authors:** Arisa Kita, Sena Yamamoto, Yuki Saito, Takako S. Chikenji

**Affiliations:** ^1^ Department of Anatomy, Sapporo Medical University School of Medicine, Sapporo, Japan; ^2^ Department of Plastic and Reconstructive Surgery, Sapporo Medical University School of Medicine, Sapporo, Japan; ^3^ Graduate School of Health Sciences, Hokkaido University, Sapporo, Japan

**Keywords:** cellular senescence, senescence-associated secretory phenotypes (SASP), woundhealing, aged-skin, diabetic skin

## Abstract

Cellular senescence is a biological mechanism that prevents abnormal cell proliferation during tissue repair, and it is often accompanied by the secretion of various factors, such as cytokines and chemokines, known as the senescence-associated secretory phenotype (SASP). SASP-mediated cell-to-cell communication promotes tissue repair, regeneration, and development. However, senescent cells can accumulate abnormally at injury sites, leading to excessive inflammation, tissue dysfunction, and intractable wounds. The effects of cellular senescence on skin wound healing can be both beneficial and detrimental, depending on the condition. Here, we reviewed the functional differences in cellular senescence that emerge during wound healing, chronic inflammation, and skin aging. We also review the latest mechanisms of wound healing in the epidermis, dermis, and subcutaneous fat, with a focus on cellular senescence, chronic inflammation, and tissue regeneration. Finally, we discuss the potential clinical applications of promoting and inhibiting cellular senescence to maximize benefits and minimize detrimental effects.

## 1 Introduction

Cellular senescence is induced by multiple stresses, resulting in irreversible cell cycle arrest. Cellular senescence occurs in response to various triggers, including critically short telomeres, oncogenic signaling, genotoxic damage, mechanical stress, oxidative damage, nutrient imbalance, mitochondrial damage, and viral or bacterial infection. Senescent cells play a pivotal role in tissue homeostasis and pathophysiology (Kuehnemann and Wiley, 2024). Cellular senescence prevents tumorigenesis (Sharpless and Sherr, 2015) and promotes embryonic development (López-Otín et al., 2013) as well as tissue regeneration and repair (Jun and Lau, 2010; Demaria et al., 2014; Saito et al., 2020; Saito and Chikenji, 2021). Moreover, senescent cells induce pathological conditions that delay wound healing and cause excessive fibrosis, such as chronic inflammation. An important feature of senescent cells is the senescence-associated secretory phenotype (SASP), a complex mixture of pro-inflammatory cytokines, chemokines, growth factors, and proteolytic enzymes that broadly affect the surrounding environment through autocrine, juxtacrine, and paracrine effects (Admasu et al., 2021). The composition of SASP is highly heterogeneous and driven by the cell type-specific activation of innate immunity signaling pathways (e.g., cyclic GMP–AMP synthase (cGAS)–stimulator of interferon genes (STING), toll-like receptors (TLRs), and nucleotide-binding oligomerization domain (NOD)-like receptor (NLR) family pyrin domain containing (NLRPs)), mechanistic target of rapamycin complex 1 (mTORC1), and transcription factors (e.g., nuclear factor-kappa B (NF-κB), choline-binding proteins (CBPs), and GATA-binding protein 4). SASP factors have diverse effects, such as induction of pro-inflammatory/inhibitory responses, extracellular matrix (ECM) synthesis/degradation, cell proliferation/inhibition, tumorigenesis inhibition, tumor progression, metastasis, and treatment resistance (Davan-Wetton et al., 2021; Sikora et al., 2021; Martinez-Outschoorn et al., 2014; D’Ambrosio and Gil, 2023). Although senescence-associated β-galactosidase (SA-β-gal), p16, p21, p53, and senescence-associated heterochromatin foci (SAHFs) (chromatin remodeling) are generic biomarkers for senescent cells (Wang and Dreesen, 2018), a senescent cell-specific marker has not been identified. This may be due to the complex phenotype, which reflects a highly heterogeneous senescence program (Hernandez-Segura et al., 2018; Wang and Dreesen, 2018).

The skin is the most visible organ in the body and serves as a physical barrier against harmful microbes and toxins, while also shielding us from the effects of ultraviolet radiation. Various skin stressors induce cell senescence, and their beneficial functions in the wound healing process have been reported in previous studies (Jun and Lau, 2010; Demaria et al., 2014). Senescent cell burden is observed in aging or diabetic skin, which may lead to delayed wound healing, scar formation, and aesthetics. Here, we review cell senescence during wound healing in normal, aged, and diabetic skin tissues.

## 2 Role of cellular senescence in normal skin repair

Typically, wound healing involves dynamic and interactive stages, including (i) hemostasis, (ii) inflammation, (iii) proliferation, and (iv) remodeling, which partially overlap (Singer and Clark, 1999; Demaria et al., 2015). Senescent cells are crucial for wound healing and contribute favorably to wound healing responses, including the promotion of ECM deposition and epithelialization, as well as regulation of tissue remodeling, fibrosis, and inflammation (Jun and Lau, 2010; Hiebert et al., 2018; Shvedova et al., 2022). Demaria et al. developed a BAC transgenic mouse model, known as p16-3MR, to enable the detection, isolation, and selective elimination of senescent cells in living animals. Senescent fibroblasts and endothelial cells were present at wound sites a few days after skin injury, and the elimination of these senescent cells delayed wound closure, with a peak delay at 6 days after wounding. These wound-associated senescent fibroblasts enhance optimal wound healing by secreting platelet-derived growth factor (PDGF-A), a SASP factor that promotes myofibroblast differentiation and accelerates wound closure (Demaria et al., 2014). Remarkably, senescent cells present during wound healing are transient in fibroblasts (Demaria et al., 2014; Kita et al., 2022) and keratinocytes (Ritschka et al., 2017). Senescent keratinocytes that are transiently exposed to SASP factors show increased expression of stem cell-related genes, including CD34, Lrig1, and Lgr6, and skin regenerative capacity, whereas prolonged exposure to SASP factors causes subsequent cell-intrinsic senescence arrest to counter the continued regenerative stimuli (Ritschka et al., 2017). Communication network factor 1 (CCN1)/cytochrome P450 61 (CYP61) are matricellular proteins that are dynamically expressed at sites of wound repair, and they can induce fibroblast senescence through cell adhesion receptors integrin α6β1 and heparan sulfate proteoglycans (Jun and Lau, 2010). CCN1-induced senescent fibroblasts accumulate in the granulation tissues of healing cutaneous wounds and express antifibrotic genes (Jun and Lau, 2010). In addition, highly concentrated trehalose induces SA-β-gal activity in fibroblasts via the CDKN1A (p21) pathway, which upregulates dermapontin, fibroblast growth factor 2 (FGF2), epiregulin, vascular endothelial growth factor (VEGF), and angiopoietin-2, leading to angiogenesis and keratinocyte proliferation, thus promoting repair at a living skin equivalent (Muto et al., 2023). The induction of fibroblast senescence via nuclear factor erythroid 2-related factor 2 (Nrf2) activation and plasminogen activator inhibitor-1 (PAI-1) upregulation results in the deposition of senescence-promoting ECM by fibroblasts, leading to reduced scar formation, rapid skin wound epithelialization, and skin tumorigenesis (Hiebert et al., 2018).

## 3 Role of cellular senescence in wound healing in aged skin

### 3.1 Functional abnormalities and phenotypes of cells in aged skin

Aging is a biological process that manifests systemically in an organism, and it is influenced by an individual’s genes, environmental factors, and lifestyle. Skin aging is promoted by both intrinsic factors (resulting from physiological processes) and extrinsic factors (such as exposure to ultraviolet radiation and pollutants) (Wang and Dreesen, 2018). In aging skin, a functional decline occurs in stem cells, such as epidermal stem cells (ESCs), hair follicle stem cells (HFSCs), and melanocyte stem cells, leading to skin thinning, vulnerability, and impaired wound healing (Ashcroft et al., 2002; Hsu et al., 2014; Liu et al., 2022). Various differentiated cells, including keratinocytes, fibroblasts, immune cells, and melanocytes, also undergo functional decline, resulting in the manifestation of the characteristic features of aging skin (Wang and Dreesen, 2018; Chambers and Vukmanovic-Stejic, 2020).

When the differentiation ability of keratinocytes declines, the epidermis undergoes atrophy, which is associated with degenerative processes in all layers and a diminished capacity to retain moisture (Parrado et al., 2019). In older individuals, keratinocyte-secreted interleukin (IL)-1α is increased, potentially contributing to sustained inflammation (Okazaki et al., 2005). In a non-invasive proteomic analysis of human epidermal proteins, several factors involved in inflammation, including alpha-1-acid glycoprotein 1, which is implicated in the transport of endogenous ligands related to inflammation, were found to be upregulated in aged humans (Ma et al., 2020). Basal keratinocytes, which reside in the basal layer and serve as the foundation for epidermal formation, have an uneven size and shape. This leads to the flattening of the dermal papillae, which is the junction between the dermis and epidermis, rendering them more susceptible to horizontal shear forces (shear stress) on the skin surface (Ding et al., 2021). Fibroblasts, which are involved in the formation of dermal papillae, gradually lose their inherent ECM expression characteristics and begin to exhibit adipogenic properties (Salzer et al., 2018). Fibroblasts produce the ECM of the entire skin. However, decreases in fibroblasts (due to reduced proliferative capacity) and ECM density resulting from increased matrix metalloproteinase (MMP) expression (particularly MMP-1, MMP-3, and MMP-9) leads to the loss of skin strength and elasticity, manifesting as wrinkles, sagging, and vulnerability (Russell-Goldman and Murphy, 2020). In addition to these organic changes, the immunological barrier function of the skin is altered in the aged skin. The key players in immunological barrier function include keratinocytes, monocyte-derived cells (Langerhans cells, dermal dendritic cells, and macrophages), T-resident memory cells, and mast cells. The antigen-presenting capacity of keratinocytes and the secretion of antimicrobial substances diminish with age. Langerhans cells are decreased in older individuals, whereas dendritic cells exhibit reduced migration, phagocytic activity, and diminished T-cell stimulatory capacity. The number of mast cells increases in older individuals, which may be related to excessive tissue inflammation (Cumberbatch et al., 2002; Grolleau-Julius et al., 2008; Gunin et al., 2011; Chambers and Vukmanovic-Stejic, 2020; Sochorová et al., 2023).

### 3.2 Accumulation of senescent cells in aged skin and implications for wound healing

Cellular senescence plays a crucial role in wound healing and is a driving force for the manifestation of the aging phenotype of the skin (Wlaschek et al., 2021; Shin et al., 2023). Senescent fibroblasts, melanocytes, and keratinocytes that accumulate in aging skin exhibit typical senescence features, such as increased expression of p16, p21, and p53, elevated activity of SA-βgal, diminished expression of nuclear lamin-B1, and extranuclear diffusion of high mobility group box 1 (HMGB1) (Wang and Dreesen, 2018; Dańczak-Pazdrowska et al., 2023). Keratinocytes are continually turned over by desquamation, which usually prevents the accumulation of senescent cells. However, in the aged skin, keratinocytes with decreased laminB1 expression and increased p16 expression are present close to the last nucleated differentiated strata (Sochorová et al., 2023). Repetitive ultra-violet B (UVB) stimulation promotes the accumulation of senescent keratinocytes (Chambers and Vukmanovic-Stejic, 2020; Bauwens et al., 2023). Accumulation of senescent fibroblasts in the skin correlates with aging and UVB-induced senescent keratinocyte accumulation (Ressler et al., 2006; Dańczak-Pazdrowska et al., 2023). Senescent fibroblasts exacerbate the inflammatory phenotype of the tissue through the expression of SASP factors such as IL-6 and IL-8 via NF-κB, (Meyer et al., 2017; Pilkington et al., 2021), leading to the induction of melanocyte differentiation through stromal-epithelial interactions promoted by stromal cell-derived factor 1 (SDF-1) deficiency, ultimately resulting in senile pigmentation (Yoon et al., 2018). p16-Positive melanocytes represent a significant population of senescent cells in the lesions associated with aged and photodamaged skin and hinder basal keratinocyte proliferation and contribute to epidermal atrophy *in vitro* (Victorelli et al., 2019). The aging immune system interacts with senescent fibroblasts and keratinocytes, thereby contributing to the physical and immunological vulnerability of the skin (Boren and Gershwin, 2004; Agrawal et al., 2009; Pilkington et al., 2021). There is a paucity of studies on senescent endothelial cells in the skin. miR-767, which is highly expressed in senescent skin endothelial cells and their exosomes, promotes dermal fibroblast senescence (Li et al., 2023).

In aged skin, various cellular senescence processes are intertwined, leading to the loss of rational interactions between cells (Salzer et al., 2018). This process results in excessive inflammation because of SASP factors, such as inflammatory cytokines and MMP, as well as increased reactive oxygen species (ROS) production (Ashcroft et al., 2002; Wang and Dreesen, 2018). The associated increase in the number of senescent cells with aging could be caused by a decrease in the removal rate of senescent cells (Hasegawa et al., 2023) and impaired apoptotic capacity of these cells (Seluanov et al., 2001). Various immune cells such as macrophages, neutrophils, natural killer (NK) cells, and CD4^+^ T cells are responsible for the elimination of senescent cells. The expression of the atypical major histocompatibility complex (MHC) molecule human leukocyte antigen (HLA)-E in senescent cells is induced by SASP factors, particularly IL-6, and is elevated in senescent skin cells of older individuals. Senescent dermal fibroblasts evade NK and CD8^+^ T cell responses via HLA-E expression (Pereira et al., 2019). Macrophages secrete tumor necrosis factor (TNF)-α, inducing apoptosis in senescent dermal fibroblasts in the skin and subsequently phagocytosing the dead cells. However, this action can potentially be suppressed by dermal fibroblast SASP factors (Ogata et al., 2021).

The accumulation of senescent cells with aging may contribute to delayed wound healing, and the removal of senescent cells may improve the wound healing process. p21-positive fibroblasts, which increase in response to skin injury in aged mice, persist and delay the wound healing process, owing to the delayed initiation of the proliferation phase (Jiang et al., 2020). This effect was ameliorated by local and temporary inhibition of p21 expression via siRNA (Jiang et al., 2020). In contrast, in studies on senescent keratinocytes in the wounded areas of young and aged human skin, it was observed that while the expression of p21/p53 was induced in the epidermis of the wound bed in young individuals several days after injury, it was not induced in the elderly, suggesting the suppression of beneficial cellular senescence in wound healing in the elderly, contributing to delayed wound closure (Chia et al., 2021).

## 4 Role of cellular senescence in wound healing in diabetic skin

Impairment of wound healing is a common pathological condition in diabetes, and 20%–40% of all patients with diabetes develop ulcers (Boulton, 2019). Common features of diabetic ulcers include increased inflammation and MMPs and decreased cell proliferation and migration of fibroblasts and keratinocytes (Frykberg and Banks, 2015; den Dekker et al., 2019; Li et al., 2019; Chang and Nguyen, 2021; Lobmann et al., 2002). Senescent cells are increased in patients with diabetes and diabetic animal models, especially in the adipose tissue (Kita et al., 2022). Senescent cell accumulation has also been found in diabetic complications, such as diabetic nephropathy, retinopathy, and cardiovascular disease (Oubaha et al., 2016; Xiong and Zhou, 2019; Tai et al., 2022). Several studies have reported on the contribution of senescent cells to chronic wounds and diabetic ulcers (Wilkinson et al., 2019; Wei et al., 2023; Yu et al., 2023).

In a patient with a diabetic ulcer, histological analysis showed that the expression of SA-β-gal and p16 was upregulated in the dermis (Wei et al., 2023). RNA-seq analysis of whole-skin biopsies from patients with diabetic ulcers revealed increased senescence and SASP markers, including CDKN1A, C-X-C motif chemokine ligand 8 (CXCL8), insulin-like growth factor binding protein 2 (IGFBP2), IL1A, MMP10, serine protease inhibitor clade E member 1 (SERPINE1), and TGF-A (Yu et al., 2023). These studies suggest that senescence is a mediator of diabetic ulcer pathogenesis; however, the cell type involved in this pathology remains unclear.

In diabetic rat-derived dermal fibroblasts, the expressions of common senescence markers (SA-β-gal, γH2AX, p53, and p21) are upregulated (Bitar et al., 2013). The study also showed that senescent fibroblasts derived from diabetic rats reduced their response to growth factors such as PDGF, insulin-like growth factor-1 (IGF-1), and EGF, thereby inhibiting their proliferative and migratory capacities (Bitar et al., 2013). Another study showed that mouse skin-derived fibroblasts induced senescence in a high-glucose environment, and senescent fibroblasts exhibited ferroptosis resistance, resulting in senescent cell accumulation (Wei et al., 2023). In addition, senescent macrophages are involved in diabetic ulcers. Wilkinson et al. reported that a diabetic mouse model had a large population of p16-positive macrophages in the wounds (Wilkinson et al., 2019). The study also found that senescent macrophages increased the expression of CXCL2 as a SASP factor and that CXCL2 induced senescence in dermal fibroblasts via C-X-C chemokine receptor type 2 (CXCR2), which acts as a profibrotic senescent cell by increasing the expression of COL1A1, COL3A1, and MMP2 (Wilkinson et al., 2019). These studies suggest that senescence in both fibroblasts and macrophages is involved in the pathogenesis of diabetic ulcers.

The skin is predominantly accompanied by a subcutaneous layer of adipose tissue (subcutaneous white adipose tissue: sWAT) throughout most parts of the body. In addition to sWAT, the skin has distinct layers of adipose tissue under the reticular dermis called dermal white adipose tissue (dWAT) (Driskell et al., 2014).

Both sWAT and dWAT play important roles in wound healing (Schmidt and Horsley, 2013). sWAT contributes to wound healing by regulating adipocyte precursor proliferation and mature intradermal adipocyte repopulation in the skin after wounding (Schmidt and Horsley, 2013). In addition, the inhibition of adipogenesis by peroxisome proliferator-activated receptor γ (PPARγ) inhibitors impairs wound healing (Schmidt and Horsley, 2013). In addition, in A-ZIP mice (which lack WAT and serve as a model of lipoatrophic diabetes), fibroblast growth is reduced during wound healing (Schmidt and Horsley, 2013). sWAT ablation using AdipoqCre has also been reported, and Cre-inducible diphtheria toxin receptor (iDTR) mice showed impaired wound healing (Shook et al., 2020). Furthermore, adipocytes at the wound sites migrate to the wound bed and transdifferentiate into myofibroblasts to promote wound healing (Shook et al., 2020). These results suggest that dWAT is required for the presence of fibroblasts in wounds.

sWAT contributes to wound healing, and wound healing time increases when sWAT is removed (Hu et al., 2016). We previously investigated the role of sWAT in diabetic wound healing (Kita et al., 2022) and found that the transplantation of sWAT derived from diabetic mice into non-diabetic mice impaired wound healing. We also investigated the role of sWAT senescence in diabetic wound healing. The expression of SASP factors during the wound-healing process showed dynamic changes in the sWAT of non-diabetic mice; however, these changes were small in the sWAT of diabetic mice. We also found that mesenchymal cells in sWAT were the main population of cells that exhibited senescence. In sWAT from non-diabetic mice and healthy patients, senescent mesenchymal cells were abundant in the early phase of the wound; however, in sWAT from diabetic mice and patients, senescent cells gradually increased after the wound. Finally, we showed that different components of SASP factors from sWAT affect wound closure, and although non-diabetic sWAT-derived SASP factors promote fibroblast migration, diabetic sWAT-derived SASP factors inhibit fibroblast migration (Kita et al., 2022). These studies demonstrated the significance of adipose tissue and its senescence in non-diabetic and diabetic skin tissues. Although there is a potential for senescence-targeted therapy for adipose tissue in the skin, its feasibility remains unclear.

## 5 Anti-senescence therapeutic interventions (molecular tools, senolytics, and senomorphics)

Therapeutic interventions for cellular senescence, known as senotherapeutics, can be categorized into two groups: senolytic and senomorphic drugs. Senolytic drugs selectively eliminate senescent cells, whereas senomorphic drugs inhibit the effects of SASP factors (Shin et al., 2023; Zhang et al., 2023). Treatment with senolytic drugs, such as the Bcl-2 inhibitors, ABT-263, and ABT-737, has been implicated in age-related skin therapy. In mouse models, senolytic treatment selectively removes senescent skin fibroblasts, thereby promoting increased collagen density, epidermal thickness, and keratinocyte proliferation while suppressing SASP, including MMP-1 and IL-6 (Kim et al., 2022). Additionally, ABT-263 treatment selectively induces apoptosis in p16-positive human senescent fibroblasts but not in normal fibroblasts and suppresses melanin production in skin co-cultured with senescent fibroblasts and melanocytes, potentially reducing skin pigmentation caused by photoaging (Park et al., 2022). The mTOR pathway has attracted considerable attention as a potential target for senomorphic drugs (Chrienova et al., 2022; Shin et al., 2023). Rapamycin, an mTOR inhibitor, significantly reduces senescent markers and SASP factors in UV-induced fibroblasts in photoaging human skin and leads to a decrease in oxidative stress (Bai et al., 2021). Rapamycin treatment inhibits stress-induced premature senescence due to the activation of the Nrf2 pathway and suppression of senescent markers, such as p16, p21, and H2AX (Wang et al., 2017). Senescence-targeting immunotherapeutics may be included among these senotherapeutics (Park and Shin, 2022). Carnosine, an endogenous dipeptide consisting of L-histidine with β-alanine, improves macrophage-mediated elimination of senescent keratinocytes and fibroblast cells under culture conditions (Li et al., 2020).

Niyogi et al. (2023) reported that the combination of ABT-737 (a BCL2 inhibitor) and FGF2 treatment promoted both the reduction of senescent cells and the migratory ability of non-senescent cells in in vitro and *ex vivo* healing models. Senomorphic drugs, such as metformin and resveratrol, promote wound healing in aged animals by downregulating the expression of p53, p21, and p16 in wound bed cells, preventing the inactivation of age-related adenosine monophosphate (AMP)-activated protein kinase (AMPK), and alleviating the inhibition of angiogenesis (Zhao et al., 2017).

Although accumulating evidence suggests that senescent cells play a role in the inhibition of wound healing in diabetes, research on the potential for targeting cellular senescence to treat diabetic ulcers is limited. Wilkinson et al. (2019) reported that blocking CXCR2 with the CXCR2 antagonist SB265610 improved wound healing in a diabetic mouse model by inhibiting macrophage senescence and inflammation.

## 6 Conclusion

**FIGURE 1:**
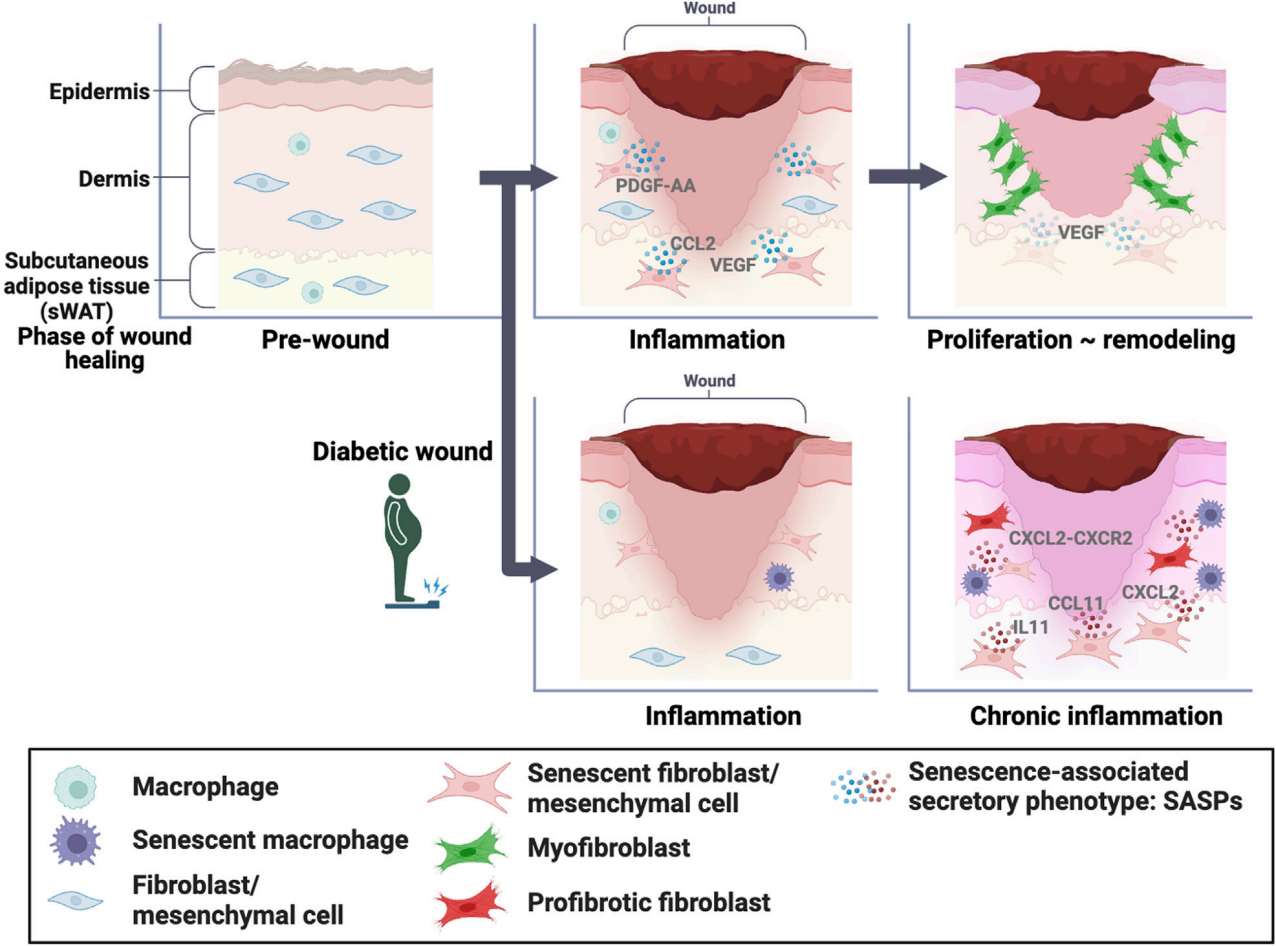
Role of senescent cells in wound healing In normal skin, senescent dermal fibroblasts and mesenchymal cells in the subcutaneous white adipose tissue (sWAT) play essential roles in wound healing through senescence-associated secretory phenotype (SASP) factors, which promote optimal wound closure through myofibroblast differentiation. Conversely, senescent fibroblasts and macrophages inhibit wound healing via CXCL2-CXCR2 signaling in diabetic skin. In addition, the accumulation of senescent mesenchymal cells in sWAT inhibits wound healing.

Cellular senescence is a state of permanent cell cycle arrest characterized by alterations in cell morphology and functionality. Senescent cells lose their division and proliferation ability but remain metabolically active and can influence their surrounding microenvironment through the secretion of inflammatory molecules and growth factors called SASPs. In normal skin, senescent fibroblasts play an essential role in wound healing by PDGF-AA secretion, which promotes optimal wound closure via myofibroblast differentiation (Figure 1). Senescent fibroblasts and macrophages inhibit wound healing via CXCL2-CXCR2 signaling in diabetes (Figure 1). Our study also reported that senescent mesenchymal cells in sWAT promote wound healing in normal skin by increasing the expression of SASP factors (Figure 1). Furthermore, we found that gradually increasing the numbers of senescent mesenchymal cells in sWAT after wounding impaired diabetic wound healing. Unfortunately, the distinguishing features of beneficial and detrimental senescent cells are still unknown; however, there is some consensus that a transient increase in the proportion of senescent cells exerts beneficial effects, and prolonged accumulation of senescent cells exerts detrimental effects.

In this review, we describe how cellular senescence is involved in both the promotion and inhibition of wound healing. However, research on therapeutics targeting senescent cells remains limited. Senotherapeutics is a promising approach for treating various diseases, and their development is expected (Raffaele and Vinciguerra, 2022). Therefore, it is important to gain an in-depth understanding of the complex roles of senescence.
